# Totipotency segregates between the sister blastomeres of two-cell stage mouse embryos

**DOI:** 10.1038/s41598-017-08266-6

**Published:** 2017-08-15

**Authors:** E. Casser, S. Israel, A. Witten, K. Schulte, S. Schlatt, V. Nordhoff, M. Boiani

**Affiliations:** 1 Max Planck Institute for Molecular Biomedicine, Roentgenstrasse 20, Muenster, 48149 Germany; 2 University Hospital Muenster, Core Genomic Facility, Albert-Schweitzer-Campus 1, Building D3, Muenster, 48149 Germany; 3 University Hospital Muenster, Centre of Reproductive Medicine and Andrology (CeRA), Albert Schweitzer-Campus 1, Building D11, Muenster, 48149 Germany

## Abstract

Following fertilization in mammals, it is generally accepted that totipotent cells are exclusive to the zygote and to each of the two blastomeres originating from the first mitotic division. This model of totipotency was inferred from a minority of cases in which blastomeres produced monozygotic twins in mice. Was this due to experimental limitation or biological constraint? Here we removed experimental obstacles and achieved reliable quantification of the prevalence of dual totipotency among mouse two-cell stage blastomeres. We separated the blastomeres of 1,252 two-cell embryos, preserving 1,210 of the pairs. Two classes of monozygotic twins became apparent at the blastocyst stage: 27% formed a functional epiblast in both members (concordant), and 73% did so in only one member of the pair (discordant) – a partition that proved insensitive to oocyte quality, sperm-entry point, culture environment and pattern of cleavage. In intact two-cell embryos, the ability of sister blastomeres to generate epiblast was also skewed. Class discovery clustering of the individual blastomeres’ and blastocysts’ transcriptomes points to an innate origin of concordance and discordance rather than developmental acquisition. Our data place constraints on the commonly accepted idea that totipotency is allocated equally between the two-cell stage blastomeres in mice.

## Introduction

One of the key goals in developmental and reproductive biology is to better understand totipotency: the capability of a single cell to produce a fertile adult organism when placed in a supportive environment^1, 2^. Totipotency in the earliest mitotic products of the fertilized oocyte, the two-cell stage blastomeres, is documented by the phenomenon of bichorionic biamniotic monozygotic (MZ) twinning in mammals. While natural MZ twinning is rare in mice^3^, it can be produced experimentally by separating the blastomeres^4^, but records of two-cell stage blastomeres forming two live-born mice have been rare. However, experiments showing that mouse two-cell embryos can compensate for the loss of one blastomere^5, 6^ led to this conjecture: If one blastomere is destroyed and the remaining blastomere can still form a mouse, then we generalize and assume that both blastomeres are totipotent. In fact, the totipotency of both blastomeres of the same two-cell mouse embryo was proven rigorously in only four reports, using the classic bisection technique. The reports showed that 1 of 26 pairs^7^, 6 of 23 pairs^8^, 9 of 26 pairs^9^ and 4 of 10 pairs^10^ were able to yield two live-borns after embryo transfer to the uterus. Two-cell embryo bisection in other species resulted in one live-born (singleton) after transfer of 22 MZ pairs in the Rhesus monkey^11^, five live-born pairs after transfer of 16 MZ pairs in the sheep^12^, and 9 live-born pairs after transfer of 77 MZ pairs in the rat^13^.

More recent attempts with refined methods could not achieve better results in mice. When 262 two-cell embryos were split, 44–83% of the cells survived the manipulation, but none of the resultant blastocyst pairs were transferred to uterus^14^. When eight twin blastocyst pairs were transferred to uterus, one pair was recovered at gastrulation^15^; this number increased to three pairs when the embryos were treated with the small molecule inhibitors of the mitogen-activated protein kinase kinase and glycogen synthesis kinase 3, known as “2i” (CHIR and PD inhibitors)^16^, prior to uterus transfer^15^. This increase in developmental capacity was ascribed to an increase in the number of *Nanog*-expressing pluripotent epiblast (EPI) cells in the blastocysts. It is possible that sufficient cell numbers might need to be present not only in the EPI but also in the other blastocyst compartments, namely the *Cdx2*-expressing trophectoderm (TE) and the *Sox17*-expressing primitive endoderm (pEnd). When the female genital tract, with its strict timing and high selective pressure that is associated with implantation, was replaced with the milder conditions of *in vitro* culture on a feeder layer, twin blastocysts attached to the feeders and formed outgrowths, but none of the sister outgrowths yielded a pair of embryonic stem (ES) cell lines^17^. When sister blastomeres were cultured directly on a feeder layer, only 1 of 6 pairs yielded a pair of ES cell lines^18^.

It appears from the records above that the totipotency of sister mouse blastomeres has been thoroughly tested in 107 (26 + 23 + 26 + 10 + 8 + 8 + 6) two-cell embryos distributed over a period of 35 years (1983–2017). Most of the separated blastomere pairs (82/107) reached the endpoint of birth, the intermediate point of ES cell derivation or gastrulation in one member of the pair but not in both. Paradoxically, there have been more mice produced by the more invasive and difficult method of somatic cell nuclear transfer (SCNT) than by the simpler method of two-cell embryo bisection. Given these records, it is almost inevitable that the question as to whether the two sister blastomeres are equally totipotent has remained open. Our data place constraints on the commonly accepted idea that totipotency is allocated equally between the two-cell-stage blastomeres in mice based on 1) a minimally detrimental manipulation of 1,252 two-cell embryos, and 2) multiple endpoints including the gene expression of sister blastomeres and the germ layer makeup of the resultant MZ blastocysts (EPI, TE, pEnd). A reconsideration of totipotency may allow the reconciliation of the different views on the biology of the first two blastomeres: The view that the sister blastomeres are inherently the same (failures to produce two mice are due to experimental damage to one blastomere, for example), and the view that the sister blastomeres are inherently different (failures to produce two mice are due to this difference)^19^.

## Results

### A robust, least-injuring method of two-cell embryo bisection that is suitable for generating MZ twin blastocysts in mice

**Figure 1:**
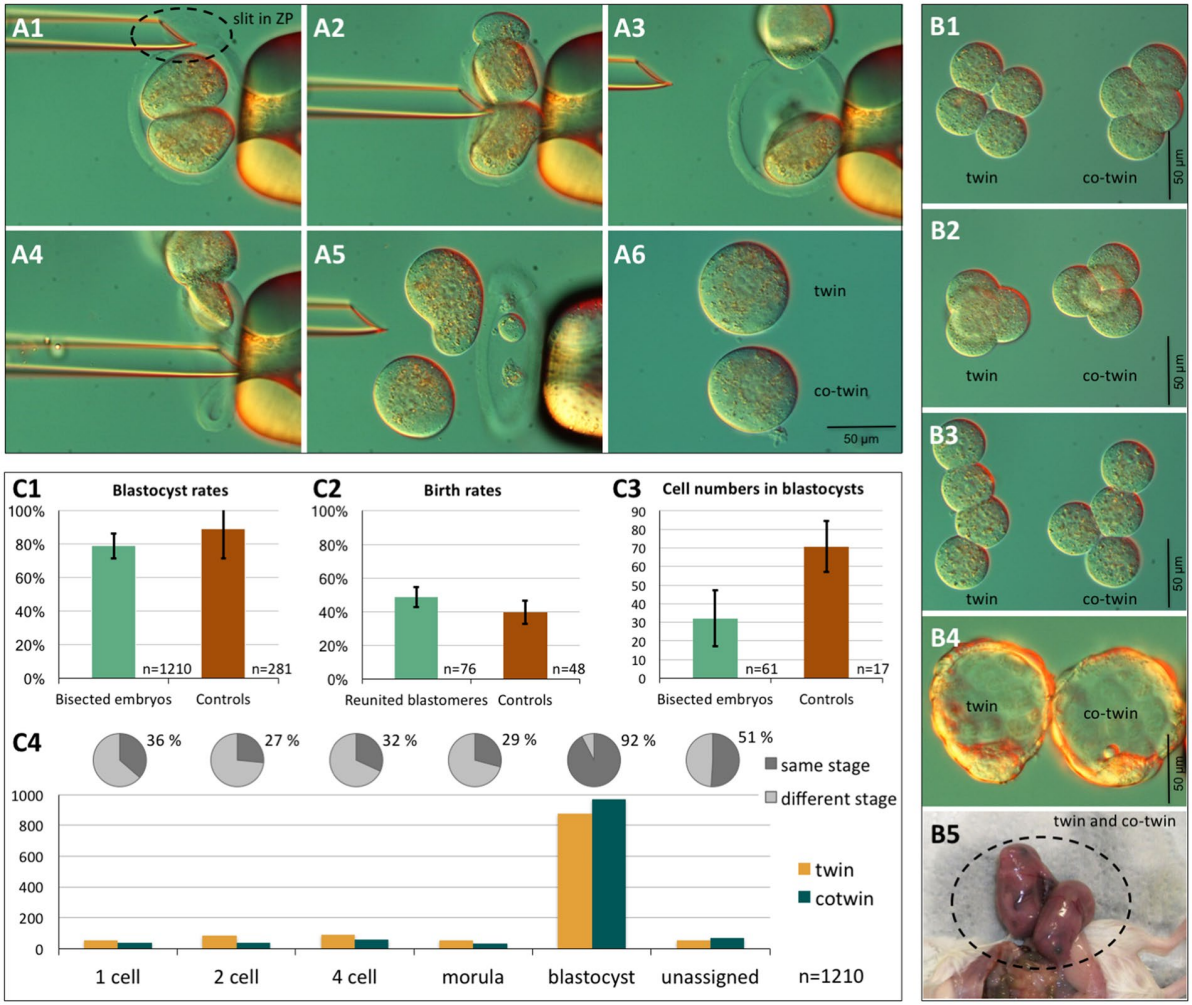
Micromanipulation and culture of two-cell mouse embryos to generate monozygotic (MZ) twins. Embryos were held in a micromanipulation medium lacking calcium and phosphate. A holding pipette (right side in the picture of series **A**) applied negative pressure to hold a two-cell stage embryo at the equatorial region, aligning its cleavage axis with the pipette’s long axis. Using a spiked beveled pipette as a bisection tool (left side in the picture), a slit was made in the zona pellucida (ZP) at the upper pole of the embryo (**A1**). The upper blastomere was pushed through the slit by gently pressing the bisection pipette against the ZP at the equator, while the pressure in the holding pipette was slightly reduced (**A2–A3**). The second blastomere was released by pressing the ZP below the equator (**A4–A5**). Separated blastomeres recovered their round shape within a few minutes after bisection (**A6**). Upon culture in KSOM(aa) medium, the sister blastomeres cleaved and developed to the four-cell stage (planar conformation, (**B1**); tetrahedral conformation, (**B2**); open chain conformation, **B3**) and further to the blastocyst stage (**B4**). When MZ blastocysts were transferred as a pair to the same uterine horn, cases were recorded in which two fetuses were found in that horn (**B5**), suggesting that at least some two-cell embryos were totipotent in both blastomeres. Monozygotic twins formed blastocysts at the same rate (**C1**), comprising half the number of cells as unmanipulated blastocysts (**C3**). After transfer to the uterus, blastocysts obtained from bisected and then immediately reunited blastomeres yielded as many fetuses as unmanipulated controls (**C2**). The error bars associated with the diagram bars are standard deviations. Twin pairs that formed two blastocysts were equally frequent among the sister blastomeres, as were twin pairs that arrested at pre-blastocyst stages (**C4**).

Blastomeres of two-cell B6C3F1xCD1 embryos were extracted one after the other through a slit made in the zona pellucida (ZP) using a beveled micropipette within 2 h of the first cleavage (Fig. 1A1–A6; Supplementary video). Dual blastomere survival was 96.7 ± 2.8% (n = 1,252 two-cell embryos; 1,210 survived bisection). Sister blastomeres developed further (Fig. 1B1–B4) when they were cultured apart, ZP-free in KSOM(aa) medium in 96-well plates with round bottoms, with blastocyst rates similar to intact blastocysts (Fig. 1C1). We refer to the developing sister blastomeres as twin and co-twin, followed through 72 h of development post-bisection, i.e. 90 h from zygote collection.

We applied the following two controls to show that our bisection method, although involving significant physical manipulation, did not *per se* compromise the developmental ability of twin and co-twin. Firstly, we reunited sister blastomeres after extraction from the ZP. Reunion succeeded (192/200) in all cases, except when the manual positioning of the blastomeres failed to put them in direct contact within 30 s, in which case the pair was excluded from the experiment to move on to the next pair. The reunited blastomeres formed blastocysts in 90.6% of the cases (174/192). After transfer to the uteri of pseudopregnant foster females, the birth rates of reunited blastomeres (37 live-borns/76 blastocysts; 10 ETs) were like the birth rates of non-bisected counterparts (19 live-borns/48 blastocysts; 10 ETs) (Fisher’s exact test, p = 0.36; Fig. 1C2). As a second control, we verified that the blastocysts developing from a single two-cell blastomere had half the total cell number as unmanipulated controls (32.1 ± 15.0, 70.8 ± 13.6; n = 20 per group; Fig. 1C3) and did not differ in the proportion of blastocyst cells positive for caspase-3 (3.2 ± 4.4, 4.1 ± 4.0; p = 0.64, t-test; n = 15 per group), which plays a dominant role in the apoptotic pathway. With the confidence of these control experiments, we performed preliminary transfers of twin and co-twin blastocysts to the uterine horns of pseudopregnant foster females to provide a proof of principle that these embryos could complete development (Fig. 1B5).

In reciprocal comparison, twin and co-twin had similar probability of reaching the blastocyst stage or of arresting at any stage before blastocyst (one-cell, two-cell, four-cell, eight-cell and compacted morula), as shown by the frequency distribution (Fig. 1C4). When a twin formed a blastocyst, the co-twin also did so 92% of the time (see pie chart insert in Fig. 1C4). A lower percentage of same-stage twins was recorded among the arrested stages. Overall, taking all 1,210 pairs together, the cleavage behavior of the twin was not distinguishable from that of the co-twin (p = 0.472; Wilcoxon signed-rank test).

### MZ twin blastocysts belong to two functional classes

The occasional completion of development by both members of a MZ twin pair (Fig. 1B5) can only show at best that some of the two-cell embryos contain two totipotent blastomeres. A reliable assessment of the actual prevalence of dual totipotency among the sister blastomeres requires a sufficiently large population of MZ twins. We used separated-reunited (reconstructed) two-cell embryos and unmanipulated two-cell embryos as controls. The latter, which arose from different oocytes of the same mother, can be considered as dizygotic (DZ) twins.

**Figure 2:**
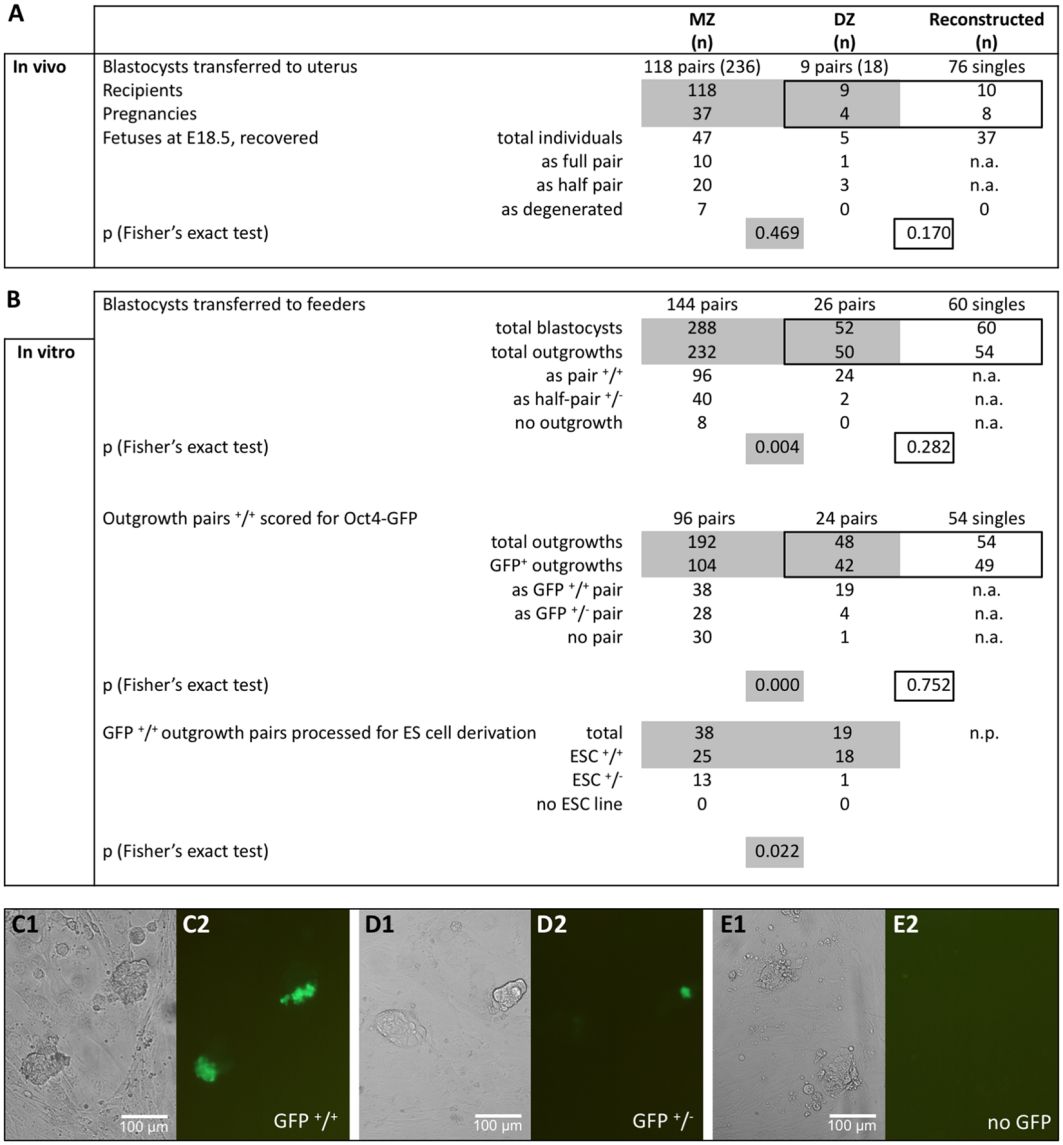
Functional analysis of MZ twin pairs. Single pairs of MZ twin blastocysts were transferred to the uteri of pseudopregnant recipients (one pair per recipient), using DZ twins and reconstructed embryos (separated-reunited blastomeres) as controls (**A**). Single pairs of MZ twin blastocysts were transferred to a layer of feeder cells to enable outgrowth formation and ES cell derivation (**B**). Statistical comparisons were conducted on the pairs of MZ and DZ twins, and on the individual embryos of DZ twins and separated-reunited blastomeres (Fisher’s exact test). Gray-shaded areas visualize the comparison of MZ and DZ twins with each other; black-framed areas visualize the comparison of DZ twins and reconstructed controls with each other. n.a., not applicable. n.p., not performed. The functional status of the outgrowths was validated by scoring the expression of Oct4-GFP (**C–E**; “1” refers to light microscopy, “2” to fluorescence microscopy).

A total of 118 pairs of MZ twin blastocysts were transferred as single pairs to the uterus, using unfertilized oocytes as carriers. These transfers resulted in 37 pregnancies – an incomplete rate that is similar to that of DZ twin pairs (Fig. 2A). MZ and DZ twins were not allowed to be delivered vaginally, unlike the separated-reunited controls, but were retrieved by caesarean section on late E18.5. Of the 37 pregnancies, 10 yielded pairs of babies, 20 yielded only one baby and 7 presented resorptions (Fig. 2A). Of the 10 MZ pairs that were given to foster mothers, 1 pair died shortly thereafter and 1 pair was cannibalized. The phenotypes of the remaining 8 MZ pairs were pursued to a limited extent, since the salient point for totipotency had already been made (full development). The MZ babies were outwardly normal, had similar weights (1.985 ± 0.292 g) as DZ twins (1.763 ± 0.285 g) and grew into fertile and outwardly healthy adults which gained weight steadily, albeit slightly less than controls (Supplementary Figure S1). The twins were assayed for physiology between the 9^th^ and the 19^th^ week of age. The metabolic rate of MZ twins (0.876 ± 0.189 mL O_2_ h^−1^ g^−1^) was lower than that of DZ twins (1.307 ± 0.286 mL O_2_ h^−1^ g^−1^).

The incomplete pregnancy rates could be due to a failure of the embryo transfer procedure or failure of the twin blastocysts to form a mature TE, which is required to support implantation. Since incomplete pregnancy rates were also observed for separated-reunited controls that were transferred as pools of 8 to the uterus (Fig. 2A), we decided to no longer transfer the blastocysts, but to monitor them *in vitro* in the so-called “outgrowth assay”. This assay shows some analogy to implantation, but can be achieved *in vitro* by the continued culture of blastocysts in complex media on a suitable substratum^18^. Outgrowth means that, as the blastocyst attaches to the feeder layer, the TE “grows out”, leaving an inner cluster of pluripotent cells (consisting of EPI cells under 2i conditions). Consequently, a total of 144 pairs of MZ twin blastocysts were transferred onto a feeder layer of fetal fibroblasts under leukemia inhibitory factor (LIF) and 2i medium in a 96-well plate, monitoring each pair over 3–4 days (Fig. 2B). Because the live-cell expression of Oct4-GFP can be used as a marker of EPI pluripotency^20^ (Fig. 2C), we recovered the embryos from B6C3F1xOG2 matings to incorporate the Oct4-GFP reporter from the OG2 mouse strain, and processed the two-cell embryos as described. Of 144 MZ twins, 96 (66.7%) attached and formed two outgrowths (the remaining MZ twins formed only one); in contrast, the majority (92.3%) of DZ twin blastocysts formed two outgrowths. Of the MZ outgrowth pairs, a minority (39.6%) presented cells expressing Oct4-GFP in both outgrowths (Fig. 2C), which is in stark variance with the DZ counterparts (79.2%), in spite of the most permissive culture conditions offered by 2i medium^16^. Most of the MZ outgrowth pairs presented cells expressing Oct4-GFP in only one (Fig. 2D) or no (Fig. 2E) outgrowths (Supplementary Figure S2). When the outgrowth pairs with dual Oct4-GFP expression were processed further, MZ and DZ twins scored similarly at ES cell derivation (>80%; Fig. 2B) and presented similar rates of chromosomal aneuploidy at passage five (15.9 ± 20.5 vs. 13.4 ± 4.1%; p = 0.66, t-test). Differences between MZ and DZ twins were not trivial effects of the mechanical bisection, as shown by examination of separated-reunited controls. These controls performed similarly to DZ twins in terms of outgrowth formation and Oct4-GFP expression (Fig. 2B).

These observations document that manipulation has a negligible effect (separated-reunited blastomeres vs. DZ twins, p > 0.05); more importantly they document the presence of two developmental classes of MZ twin blastocysts, based on the proportions of MZ pairs that are capable of proceeding in development in both members (27% in the uterus, 26% *in vitro*) as opposed to the remaining pairs that fail in either one or both members. Although the 2i medium offered the most permissive culture conditions, most MZ twins appear to differ in the predisposition and formation of their EPI.

### Twin embryo classes feature an EPI asymmetry that is independent of oocyte quality, sperm-entry point, culture environment and pattern of cleavage

We examined the MZ twin blastocysts more closely with the objective of determining whether the imbalance of EPI cells arose after the time of implantation or already existed before it. Thus, we examined the expanded blastocysts for protein markers of cell lineages, including TE and pEnd, thereby encompassing all three founder cell lineages of the blastocyst. Cell identity was determined by immunofluorescence staining and confocal imaging, according to our established method^21^ (CDX2, NANOG and SOX17 for TE, EPI and pEnd, respectively). Immunostained cells were counted manually on Z-projection images with the help of Image-J software.

**Figure 3:**
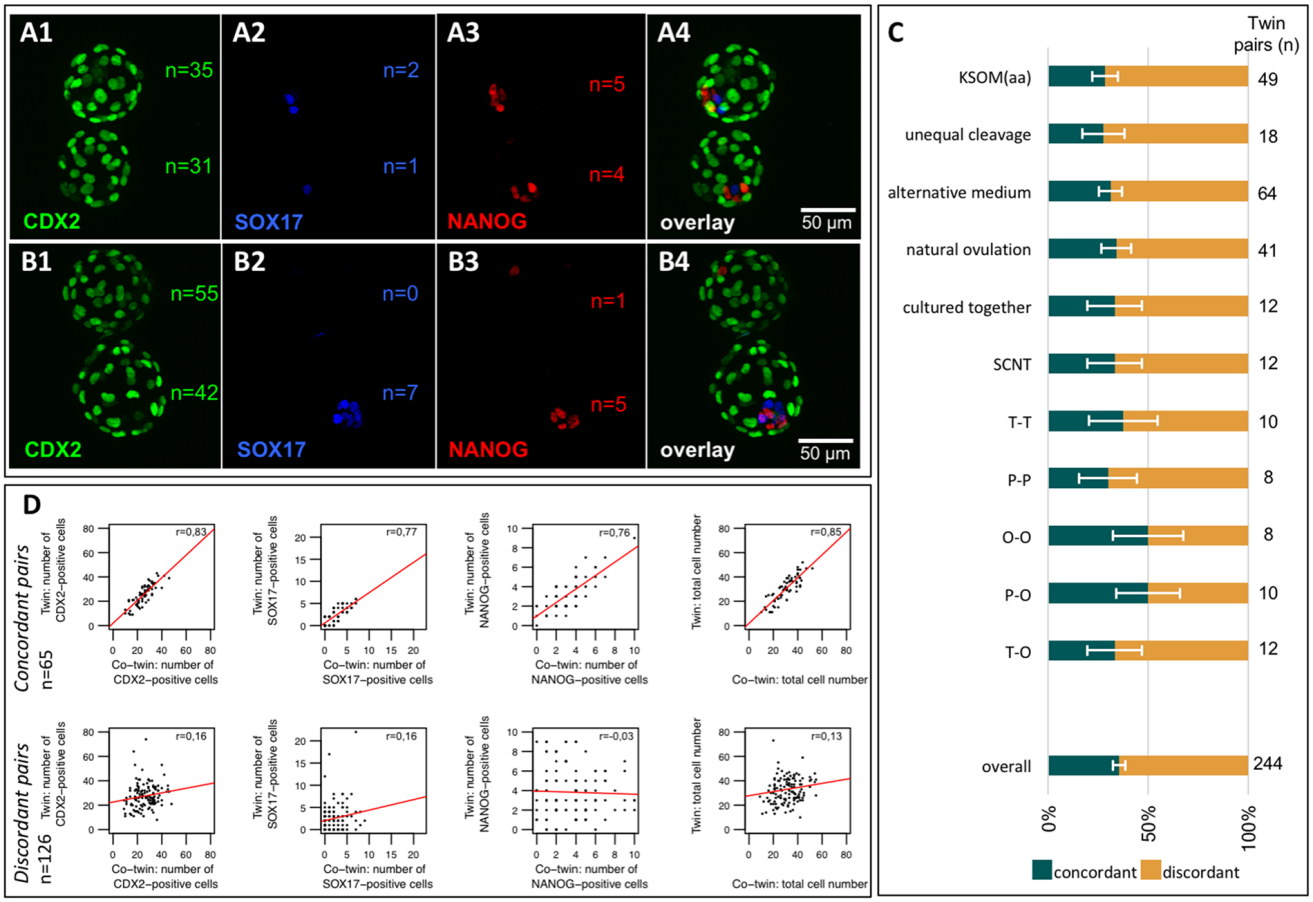
Cell lineage analysis of blastocysts. Immunoconfocal fluorescence images of representative MZ twin blastocysts, co-stained for cell lineage-specific markers: CDX2 for TE (**A1,B1**), SOX17 for pEnd (**A2,B2**) and NANOG for EPI (**A3**,**B3**). Microscope objective 20X, 0.75 N.A. Pairs were scored as either “concordant” (**A**) or “discordant” (**B**) based on the “9-2-2 criterion” (see main text). The proportions of concordant and discordant pairs were similar irrespective of culture medium, ovarian stimulation, co-culture, sperm-entry point and embryo conformation at the four-cell stage (tetrahedral, T; planar, P; open chain, O). The error bars associated with the proportions are standard deviations. (**C**). Linear correlation analysis (Pearson’s r) of the cells counted in the lineage compartments of each MZ pair shows that the main contributor to the discordance was the EPI lineage (**D**).

In line with the previous analysis of postblastocyst development, it became apparent that twin pairs were heterogeneous, featuring cases of high similarity of cell allocation between twin and co-twin as well as cases of marked diversity, as exemplified in Fig. 3A1–A4 and B1–B4, respectively. This variability of cell allocation was subjected to systematic analysis. It was essential to define the extent of variation that is present as background, since variability exists even in clonal populations of cells. The extent of background variation was extracted from the analysis of DZ twins, which arise from different oocytes of the same mother. The extent of variation among 76 DZ twin blastocysts, paired in a random manner, was quantified into 18 TE, 4 EPI, and 4 pEnd cells (median values of 38 pairs examined). Since MZ twins contain half the number of cells compared with DZ twins, we elected half of the DZ difference (18, 4, 4 cells) as the threshold that MZ twins should cross to be considered as different (9–2–2 criterion). The pairs that crossed this threshold were defined as “discordant.” Accordingly, members of each MZ pair were defined as “concordant” if the cell counts differed by no more than 9 TE, 2 EPI and 2 pEnd cells. By using this approach, we appreciated that MZ twin blastocysts can be sorted in two classes: one (36%) with concordant cell allocation, the other (64%) with discordant cell allocation, to TE, EPI and pEnd (Fig. 3C). Discordance was more pronounced in the EPI, as measured by the lower coefficient of linear correlation (Pearson’s r) of twin and co-twin (Fig. 3D). This partition into classes was proven to be stable (Fig. 3C), i.e. it was observed when twin and co-twin were derived from blastomeres of unequal sizes (shown to correlate with mitochondrial DNA copy number)^22^, when twin and co-twin were cultured in alternative media e.g. twin in medium A and co-twin in medium B (thereby perturbing the metabolic status), when twin and co-twin were next to each other while preventing contact (thereby enhancing paracrine signaling), when fertilization took place naturally (without gonadotropin stimulation) or when an effect of the sperm entry point was discounted by examining cloned blastocysts produced via SCNT (Supplementary Figure S3). Lastly, we turned our attention to intermediate cleavage stages to pursue the origin of these concordant and discordant classes. It is known that blastomeres can adopt three possible conformations at the four-cell stage of development (tetrahedral, T; planar, P; open chain, O)^23^. Therefore, we examined twin pairs at the four-cell stage, and assessed how their conformation (T-T, P-P, O-O, T-P, T-O, P-O; Fig. 1B1–B3) affected the prevalence of blastocysts with concordant vs. discordant cell allocation. Discordant blastocyst pairs arose from concordant four-cell pairs; likewise, concordant blastocyst pairs arose from discordant four-cell pairs (Fig. 3C).

These data indicate that the two embryo classes are resistant to changes of oocyte quality, sperm entry-point, environment (e.g. culture medium) or pattern of cleavage. This suggests that the variability in the two embryo classes preexist fertilization and therefore might be innate rather than acquired during development.

### EPI asymmetry also occurs within the non-bisected embryo: simultaneous tracing of *Nanog*-expressing descendants of the sister two-cell blastomeres

**Figure 4:**
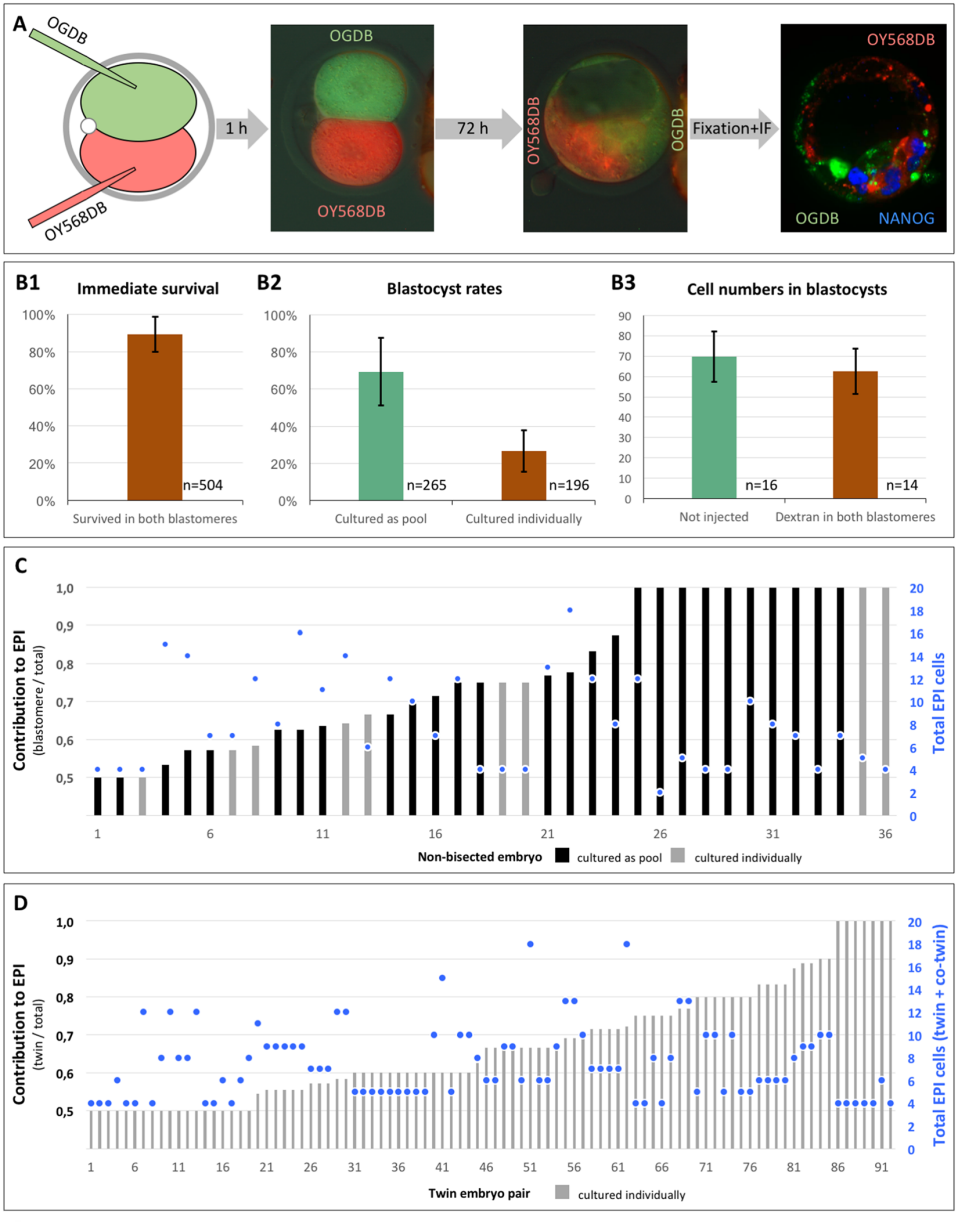
Tracing of the contribution of sister two-cell stage blastomeres to epiblast. Each blastomere in two-cell embryos were microinjected with fluorescent dextran beads of different colors (OGDB, OY568DB), followed by *in vitro* culture to blastocyst and NANOG immunofluorescence analysis (**A**). The quality of the microinjections was validated by survival rates of the blastomeres, blastocyst rates, and total cell numbers of blastocysts (**B**). The relative contribution of each two-cell blastomere to EPI (ratio from 0.5 to 1) and the absolute total number of EPI cells (•) are shown for non-bisected (**C**) and twin (**D**) embryos. Error bars in (**B**) are standard deviations. Black and gray bars in (**C**) and (**D**) indicate embryos cultured as pools and embryos cultured individually, respectively.

Since MZ twinning by separation of the sister two-cell blastomeres is not a natural process, we asked whether asymmetric behavior in the EPI compartment also would exist in sister blastomeres that remained joined together. We traced the progenies of the sister blastomeres from 504 two-cell embryos by injecting them in the cytoplasm with green (488 nm) and orange/red (568 nm) fluorescent dextran beads, being careful to use a size that would not pass through gap junctions (70 kDa) (Fig. 4A). To judge the reliability of these embryos for further study we assessed three metrics: we ensured that most of the two-cell embryos survived the dual injection (88.2 ± 8.7%), that the blastocyst rate was respectable (69.3 ± 18.3%) and that the total cell number in the labeled blastocysts was not significantly different from that of non-injected controls (62.5 ± 11.0 vs 69.9 ± 12.4 cells) (Fig. 4B).

Under these provisions we processed the labeled blastocysts for NANOG immunofluorescence using the 647 nm channel (pseudocolored in blue). EPI cells were assigned to one or the other blastomere’s progeny based on the colocalization of the NANOG signal with the green or orange/red fluorescence, using Image-J software (Fig. 4A). Qualitatively, we observed cases of either balanced or skewed EPI contribution from the sister blastomeres, including extreme cases of EPI contribution from one blastomere only. In order to achieve a quantitative dimension, we counted how many NANOG-positive nuclei were present in the green and orange/red compartments of the blastocyst. Then we calculated the proportion of cells in the larger EPI compartment relative to the total number of EPI cells (Fig. 4C). Among 36 labeled non-bisected embryos, 8 featured balanced proportions (one blastomere supplied 50–60% of the total EPI cells) and 14 featured markedly skewed proportions (one blastomere supplied more than 80% of the total EPI cells). Similar behavior of the sister blastomeres was observed in 92 pairs of MZ twins (Fig. 4D).

These data demonstrate that the two embryo classes are not induced by the bisection procedure, and that one blastomere is less (or more) potent than the other in a substantial proportion of two-cell embryos.

### Transcriptome analysis dates the origin of the two developmental classes to the first mitotic division of the zygote

We reasoned that, since the less frequent developmental class is not a rare occurrence after all (27% by function, Fig. 2; 36% by marker gene expression, Fig. 3), it should be possible to illuminate the molecular features of the two classes by subjecting a decent number of individual embryos to transcriptome profiling. We analyzed 16 individual twin and co-twin blastocysts respecting pair associations, and 8 non-manipulated blastocysts as controls, detecting 26,597 protein-coding mRNAs on Affymetrix microarrays. The microarray dataset including the raw data is available in the Gene Expression Omnibus under accession number GSE90674. A summary of these mRNAs (normalized data) is provided in Supplementary Table S1.

Transcriptomes of MZ twin and control blastocysts were highly similar (R^2^ = 0.994) overall, with 572 of 26,597 genes (2.2%) being differently expressed (p < 0.05, t-test). As judged from the *Xist* mRNA level, six of eight embryos in either group were males (XY). Gene set enrichment analysis (GSEA) of the genes differently expressed between twins and controls using GORILLA^24^ revealed enrichment in six gene ontology terms of the biological process: “negative regulation of receptor activity” (GO:2000272), “neural tube formation” (GO:0001841), “lamellipodium assembly” (GO:0030032), “negative regulation of stress fiber assembly” (GO:0051497), “tissue development” (GO:0009888) and “cell projection organization” (GO:0030030). Apoptosis-related genes *Casp3*, *Bax* and *Bcl2* were not differently expressed between MZ twin and control blastocysts (*Casp3*, p = 0.999; *Bax*, p = 0.349; *Bcl2*, p = 0.08; t-test). The very small proportion (2.2%) of genes expressed differently and their spurious or marginal relationship to processes of preimplantation development (including apoptosis) attest to the negligible effect of the micromanipulation.

**Figure 5:**
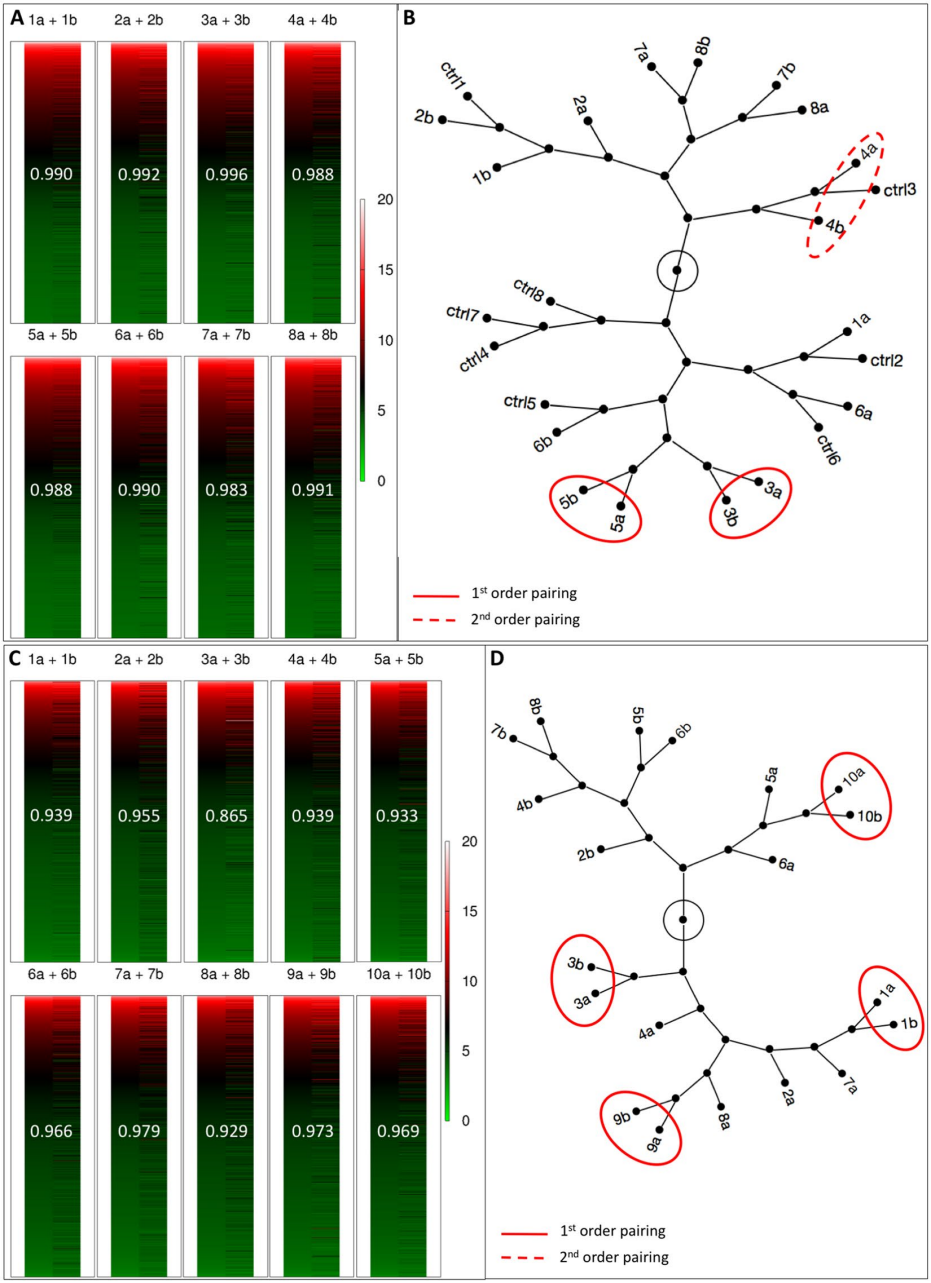
Transcriptome analysis of MZ twin pairs. Individual transcriptomes are shown as heat maps, with higher expression coded in red and lower expression coded in green, and linear correlation coefficient written on the heat maps (**A**,**C**). Constellation maps of the transcriptomes of individual blastocysts (**B**) and individual two-cell stage blastomeres (**D**) identified with both a number (for the original embryo subjected to bisection) and a letter (for the two members resulting from bisection). Unmanipulated embryos in B are labeled as controls (ctrl). The transcriptomes were subjected to hierarchical clustering analysis to see if they would return the original pair associations (e.g. 1a-1b, 7a-7b).

We next compared the MZ twins with each other, expecting to also recognize the native pair association in the transcriptomes. To harness the paired profile structure of the data, we recognized that it is not known which twin in a pair is “first” and which twin is “second,” whereby it is not possible to apply a Student’s t-test to compare “firsts” with “seconds.” Therefore, we borrowed the approach used on sister two-cell blastomeres^25^ and we calculated the ratio of each mRNA expression (E) between the pair members, always dividing the higher value by the lower value (E higher/E lower), across the eight pairs. As expected, the ratios of housekeeping mRNAs did not show any significant imbalance between twin and co-twin blastocyst (*Actb* 1.082 ± 0.063; *Eef1e1* 1.279 ± 0.174; *Gapdh* 1.046 ± 0.059*; H2afz* 1.089 ± 0.096; *Hprt* 1.250 ± 0.175*; Ppia* 1.061 ± 0.073*; Ubc* 1.142 ± 0.183; Supplementary Table S1). By contrast, a variety of other genes presented an imbalance between twin and co-twin blastocyst, resulting in average ratios >2 (1,309 genes), >5 (77 genes) and >10 (15 genes) (Supplementary Table S1). We used the coefficient of variation (CV), which is a normalized measure of the dispersion of the distribution calculated dividing the standard deviation by the mean of the ratios, to analyze the imbalance. The GSEA of the ranked list of genes (high-to-low CV) revealed the following terms: “metabolic process” (GO:0008152), “organic substance metabolic process” (GO:0071704), “primary metabolic process” (GO:0044238), “cellular metabolic process” (GO:0044237), “cell cycle process” (GO:0022402), “spermatid development” (GO:0007286), “single-organism metabolic process” (GO:0044710), “phospholipid metabolic process” (GO:0006644), “germ cell development” (GO:0007281) and “macromolecule modification” (GO:0043412) among the top ten GO terms of the biologic process. It is of note that “germ cell development” features genes expressed in the EPI, such as *Bmp4*, *Bmp8b*, *Dazl* and *Prdm14*, and the master EPI regulator gene *Nanog* presents a ratio of 2.220 ± 1.072 between twin and co-twin blastocysts. Imbalance in the EPI-related GO term “germ cell development” is consistent with the MZ pairs that could form an EPI and proceed in development in only one member as opposed to both members (Fig. 2B). Hierarchical cluster analysis of the blastocysts’ transcriptomes (Fig. 5A) identified two of eight pairs (both females, as judged from *Xist* mRNA level) in which the twins were matched with the correct co-twin, as visualized in constellation maps (see direct, i.e. first order, connections in Fig. 5B). This 25% proportion is remarkably similar to the 27 and 26% of twin pairs that succeed in the uterus and *in vitro*, respectively.

It may be argued that the twins and co-twins started out as developmentally balanced, but became imbalanced as a result of cell cycle asynchrony and/or latent damage from manipulation, to which our previous caspase-3 analysis could have been blind. Therefore, we applied the same transcriptomic pipeline to single two-cell blastomeres taken as early as the bisection could be performed without facing cell lysis (2 h after cleavage) and immediately after extraction through the slit made in the ZP. The microarray dataset including the raw data is available in the Gene Expression Omnibus under accession number GSE94050 (a summary of these data is provided in Supplementary Table S2). First we performed hierarchical cluster analysis of the blastomeres’ transcriptomes (Fig. 5C) and identified four of ten pairs in which the twins were matched with the correct co-twin (see direct, i.e. first order, connections in Fig. 5D). This proportion is not significantly different from that of correctly matched blastocysts’ transcriptomes (2/8 blastocysts and 4/10 blastomeres; p = 0.502, Fisher’s exact test). We then ranked the mRNAs based on the ratio of each mRNA between the pair members, as described for the twin blastocysts. We expected that the genes reported in the literature to be most differently expressed between sister blastomeres (e.g. *Fzd2*, *Mgat5*, *Zeb1*)^26^ would populate the upper ranks of the list; we could not verify this expectation, given ratios hardly distinguishable from 1 (e.g. *Fzd2*, 1.153 ± 0.351; *Mgat5*, 1.595 ± 1.418; *Zeb1*; 1.124 ± 0.357; t-test).

These data indicate that the two classes of two-cell embryos can be resolved on the developmental, cell lineage and global transcriptional level, and are not induced by the micromanipulation nor are they acquired later on, since they are also found immediately after the bisection. However, when it comes to the specific mRNAs involved, these appear to vary from case to case, in line with previous investigations^27^. In light of all the data above, we propose that developmental totipotency is allocated unequally – i.e. it segregates – between the two-cell-stage blastomeres in mice.

## Discussion

It is generally accepted that the totipotent cells of the mammalian life cycle are the zygote and each of the two blastomeres originating from the first mitotic division of the zygote. In fact, as our study shows, the sister blastomeres feature dual totipotency in only 30% of the two-cell embryos, as determined with a MZ twin experimental design; the remaining two-cell embryos yielded functionally discordant twins with a prominent imbalance in the EPI, leading us to propose that totipotency had segregated between the sister blastomeres. This proposal allows the reconciliation of the different views of the biology of the first two blastomeres: The view that the sister blastomeres are inherently the same (failures to produce two mice are due to experimental damage to one blastomere, for example), and the view that the sister blastomeres are inherently different (failures to produce two mice are due to this difference). Both views are correct, depending on which class of two-cell embryos is examined – the one in which the sister blastomeres are concordant, or the one in which the sister blastomeres are discordant.

One conceptual and two technical considerations were fundamental in our study. Firstly, the totipotency of the sister blastomeres did not rest on assumptions, but was tested on a considerable number of two-cell embryos: more than one thousand. Secondly, the procedure used for blastomere separation was least harmful, as shown by the nearly 100% survival of separated blastomeres and by the low incidence of apoptosis (3.2%) compared to unmanipulated embryos (4.1% in this study, 6.2% in ref. 28), as well as by the embryo reconstitution experiment. The probable explanation for this high blastomere survival is twofold: (1) The volume of the perivitelline space was increased in the slightly hyperosmotic medium used for micromanipulation (this made it easier to introduce a slit into the ZP without touching the blastomeres); and (2) the composition of micromanipulation medium relinquished phosphate, which was previously shown to be detrimental to embryo development (see Materials and Methods). Thirdly, the analysis of totipotency was based rigorously on matched sister blastomeres, i.e. respecting the pair associations, and without external cues such as the shape of the ZP^29, 30^. The experimental setting was instrumental to secure this information. Given the necessity to match the data of twin and co-twin while preventing their mixing or, even worse, their fusion, we used a single ZP-free embryo culture system in 96-well plates. A similar approach to embryo culture was used to study human MZ twin embryos on the EmbryoSlide^®^^31^.

We surgically transferred single pairs of blastocysts to the uterus of pseudopregnant recipients without using other embryos as carriers, to ensure unambiguous identification of the MZ twins and rule out rescuing effects, for thorough testing of totipotency. Mice – although a polytocous species – can support single embryo transfers^32–34^. Of the cases in which the recipients became pregnant, the frequency of two twin babies recovered at late day 18.5 was 26%. These babies were overtly normal although they gained slightly less weight and had a lower adult metabolism compared to the DZ twins. The outcome of surgical embryo transfer has multiple components e.g. a biological component related to the intrinsic quality of the embryos and receptiveness of the uterus, and a technical component related to the surgical procedure. This makes it difficult to interpret the results when, for example, pools of non-manipulated embryos were transferred to the uterus and half^35^ up to 70%^36, 32^ of them failed to yield live fetuses. To put any concerns to rest, we discontinued the embryo transfers and placed the twins – individually one per well – in the most permissive environment offered by LIF + 2i medium on feeders^16^ (both twins in the same well for photographic documentation). Under these conditions, nearly all control embryos ‘implanted’ on feeders and built functional EPI colonies expressing Oct4-GFP, compared to 66.7% (implantation) and 26.4% (Oct4-GFP positive EPI colonies), respectively, of the MZ twin pairs. Importantly, the manipulated and the non-manipulated controls performed equally well, thereby discounting a technical explanation for the low performance of MZ twins. Clearly, even without a uterus and its window of implantation that selects against blastocysts that have few cells or that develop slowly, most MZ twins were still not able to catch up and deploy functional TE and functional EPI. Derivative ES cells were predominantly euploid (85–87%), within the expected range for normal ES cell lines^18^. These functional data exposed one class (≈30%) of equally performing twins, and the other class (≈70%) of differently performing twins. We named these twins “concordant” and “discordant”.

How the discordant class of twins and particularly the different EPI behavior originates inside of the same MZ pair can have several possible explanations. The explanations would generally fall into two categories: innate or acquired origin. The cytoplasm of the oocyte, for example, does not divide equally into daughter blastomeres after the first cleavage, with up to a 10% difference in volume between the smallest and the largest blastomere^22^. These differences of blastomere size correlate positively with the number of DNA copies present in mitochondria^22^, whose unequal segregation might be implicated in the fate of blastomeres. Thus, we kept track of the size of the sister blastomeres, selecting and scoring an additional series of MZ twins in which the initial blastomeres differed in size by more than 5%. However, the blastocyst imbalance (30/70%) was still observed. It was also proposed that, in most mouse embryos, the point where the sperm originally entered the oocyte marked the blastomere that cleaved faster and contributed preferentially to the embryonic part (ICM) and, *vice versa*, progenies of the other unmarked blastomere contribute predominantly to the abembryonic pole^37^. Indeed the sperm tail can be found in one of the sister blastomeres^38^. Another study found that the first dividing blastomere of a two-cell embryo divided meridian relative to the second polar body, and the second dividing blastomere divided equatorially, giving rise to a tetrahedral conformation; in the majority of cases, the first blastomere contributed mostly to the embryonic pole and the second to the abembryonic pole of the blastocyst^39^. These observations promoted the view that the fate of the two-cell blastomeres is determined during the first cleavage (prepatterning), although this view was not shared by all^29, 40, 41^. Given the above observations and conflicting interpretations, it was logical for us to posit that the discordance between twin and co-twin could stem from the sperm entry point being inherited by the progeny of one blastomere, but not by progeny of the sister blastomere, and that the two classes of concordant and discordant MZ blastocyst pairs could be a manifestation of concordant (e.g. T-T) or discordant (e.g. T-O) conformations at the four-cell stage. We examined cloned embryos, which have no sperm entry point and, thus, should exhibit no intra-pair lineage imbalance, to test for this possibility. However, the blastocyst imbalance (30/70%) was still observed. Intermediate assessment of the twins at the four-cell stage revealed that the proportions of blastocyst pairs with concordant vs. discordant lineage compositions are also independent of the tetrahedral, planar or open chain conformation adopted by the twins at the four-cell stage, suggesting that the concordance/discordance balance is not easily perturbed after the two-cell stage. It has been suggested that hormone treatment for superovulation^42, 43^ or different culture media^21^ would influence the quality of embryos obtained, casting environmental effects on development. However, using embryos from unstimulated ovarian cycles, using an alternative culture medium or culturing the twins side by side (thereby enhancing paracrine signaling) did not alter the partition (30/70%) of the two classes. Since the partition and the EPI asymmetry were observed in bisected embryos, which are not natural, one was left to wonder if the sister blastomeres would have behaved differently if they had not been separated from each other. It is impossible to generate MZ twins from the two-cell stage without physically separating the sister blastomeres. Therefore, we applied a cell tracing system^44, 41^ by which we could distinguish the progenies of the sister blastomeres without taking them out of the ZP; we combined said system with immunofluorescence for NANOG. Embryos with prominent EPI asymmetry were still observed, thereby reproducing independent findings^45^ in which 7 of 25 embryos had an EPI contribution skewed (>70%) toward one blastomere, and very skewed (>80%) in 2 of 25 embryos. These data^45^ and our own add strength to the notion that, during normal development, there are two-cell embryos in which one blastomere is much less (or more) totipotent than the other (contribution to EPI 20/80 instead of 50/50).

The data discussed so far suggest strongly that the two-class partition is innate as opposed to acquired. Acquired variability, by definition, is null at t_0_ and increases during embryonic development. We used class discovery clustering of the transcriptomes to see whether the frequency of correctly assigned MZ pairs is higher in the blastocysts than in the bisected two-cell embryos. The frequency of correctly matched twin and co-twin blastocysts as revealed by cluster analysis was 25% (2 of 8 pairs), consistent with the size of the classes defined by function (Fig. 2) and by marker gene expression (Fig. 3). Unfortunately, it is not possible to compare our gene expression data with those of other studies, simply because side-by-side transcriptome analysis of MZ twin blastocysts of the mouse has never been reported. Although one record exists in bovine^46^, the twins of the bovine study are not comparable with ours, as they were generated by cutting the blastocysts and not by separating the two-cell stage blastomeres. In reciprocal comparison, the transcriptomes of sister MZ blastocysts differ mainly for gene ontologies of the metabolic family, consistent with the view that metabolic heterogeneity sets the stage for differences of cell differentiation^47^. When we examined the two-cell stage blastomeres taken immediately after bisection 2 h after cleavage, the frequency of correctly matched sister two-cell blastomeres (4 of 10 pairs) was similar to that of correctly matched twin and co-twin blastocysts (2 of 8 pairs). Taken together, these observations place the origin of the two functional classes of blastocysts in the early blastomeres or in the oocyte, although the analysis of blastomeres’ transcriptomes probably included a few embryos that would not have formed blastocyst.

Coexistence of two-cell stage blastomere pairs with similar or different gene expression had been noticed previously^26^, but was not ascribed to classes of embryos, rather to random partitioning of transcripts between the sister blastomeres^27, 48^. Indeed, there have been various studies, each reporting its own set of genes whose mRNAs were differently expressed between the sister two-cell stage blastomeres. However, the overlap across the studies appears to be quite small e.g. 3 genes were common to a set of 561genes (Data S1)^49^ and a set of 46 genes (Table S1)^26^. Furthermore it is uncertain how closely the mRNA asymmetry may correspond to an asymmetry of the final gene product e.g. protein.

The fact that blastomere pairs with similar or different gene expression have been observed before in different studies suggests that a biological factor upstream of the developmental stimulus, mode of cleavage and environmental cues could be responsible for the difference between sister blastomeres. The variable orientation of the first mitotic plane^27^, for example, could produce blastomeres of different compositions if a gradient – whether of nucleic acid or protein – existed in the ooplasm. Such a gradient has been described for LEPTIN and STAT3 in mouse and human oocytes^50^. Western blot analysis of bisected sheep oocytes revealed that specific maternal proteins, including NANOG, are distributed asymmetrically^51^. In mice, however, *Nanog* is not transcribed in early embryos until the 8-cell stage^52^, thereby excluding the possibility that a different maternal legacy of *Nanog* mRNA in the sister two-cell blastomeres would account for the discordant sizes of the EPI compartments of the MZ blastocysts.

In summary, our single embryo analysis places constraints on the commonly accepted idea that totipotency is allocated uniformly between the two-cell-stage blastomeres in mice, showing that, contrary to previous assumptions, only some – not all – pairs of sister blastomeres are dually totipotent as measured by MZ twinning. This can explain that the historically incomplete birth rates of MZ twins^7–10^ were not a technical problem, but a biologic barrier. This conclusion has three implications. Firstly, estimating the developmental success of one blastomere based on the gene expression of its companion blastomere^53, 54^ will only work for some of the two-cell embryos. Secondly, the search for molecular signatures of totipotency should not rely on a contrast between zygotes and two-cell embryos, on the one hand, and stages past the two-cell stage on the other hand, since the two-cell embryos are a mixed population of embryos either with or without dual blastomere totipotency. Finally, although studies suggested that differentially localized epigenetic factors appear at the four-cell stage^49, 55–57^, results presented here indicate that inter-blastomere diversification had already emerged upon the first mitotic division of the oocyte.

## Materials and Methods

### Ethics statement

All mice were maintained in individually ventilated cages in the animal facility of the MPI Münster, with a controlled temperature of 22 °C, a 14/10 h light/dark photoperiod and free access to water and food (Harlan Teklad 2020SX). Experimental procedures followed the ethical guidelines of the European Laboratory Animal Science Associations. On the local regulatory level, mice were used for experiments according to the license issued by the Landesamt für Natur, Umwelt und Verbraucherschutz of the state of North Rhine-Westphalia, Germany, in accordance with the procedures laid down in the European Directive 2010/63/EU (license number 84-02.04.2016.A229).

### Collection of mouse zygotes

Six- to eight-week-old B6C3F1 females were primed with 10 I.U. each pregnant mare serum gonadotropin (Intergonan, Intervet) and human chorionic gonadotropin (Ovogest, Intervet) injected intraperitoneally 48 h apart, and then mated to CD1 or OG2 stud males. On the morning of the vaginal plug, the cumulus-oocyte complexes were recovered from the oviducts at 10 am, dissociated in hyaluronidase (50 I.U./mL in Hepes-buffered CZB medium, HCZB) and cultured in 500 µL of KSOM(aa) medium in a four-well Nunc plate without oil overlay, at 37 °C under 6% CO_2_ in air, until splitting the next day.

### Production of cloned mouse embryos

B6C3F1 oocytes were deprived of the chromosomal spindle and transplanted with cumulus cells of the same strain. Enucleation, SCNT, activation and *in vitro* culture of the reconstructed oocytes took place in α-MEM medium, as described previously^20^.

### Splitting of two-cell embryos and culture to blastocyst stage

Two-cell embryos arising in the time frame from 6 to 7 am were removed from culture to perform splitting from 8 to 9 am. These embryos were transferred in groups of 12 to a micromanipulation drop on the stage of a Nikon Eclipse TE2000-U inverted microscope fitted with Nomarski optics and holding and bisection needles in place. The micromanipulation medium consisted of 0.2 mM D(+) glucose, 0.2 mM pyruvate, 10 mM lactate, 0.5% w/v BSA, in 0.9% w/v sodium chloride, in place of the commonly used calcium-magnesium-free phosphate-buffered saline whose phosphate was shown to be detrimental to embryo development^58^. We made the medium slightly hypertonic by using 95% of the water volume. The bisection needle was a TransferTip needle operated by a CellTram Vario (Eppendorf). The two-cell embryo was rotated using the holding and bisection needle to align the cleavage plane with the common axis of the two pipettes. The two-cell embryo was firmly held in place with the holding pipette by applying negative pressure (suction). The TransferTip was used to first make a slit in the ZP at one pole and then to press the ZP gently at the equatorial region, causing one blastomere to be squeezed out. Suction in the holding pipette was reduced. The other blastomere came out by performing the same procedure, except that the ZP was pressed below the equatorial region. When all 12 embryos had been bisected, which normally took 8–10 min, the pairs of blastomeres were collected and transferred to the 96-well plate (round bottom) using a bent (≈60 degrees) mouth-operated pipette with flame-polished tip. The individual blastomeres were cultured in 75 µL medium per well for 72 h from the time of splitting (KSOM(aa) for fertilized embryos, α-MEM for cloned embryos). When the response of fertilized embryos was tested in media other than KSOM(aa), these media were obtained from different companies: Single Step Medium (Irvine Scientific); Continuous Single Culture (Irvine Scientific); SAGE 1-Step (Origio); GM501 with Gentamicin and Phenol Red (Gynemed); and G-TL (Vitrolife). For culture of the MZ twins next to each other, we pushed the second blastomere half-way through the slit in the ZP, but then allowed it to return back and, subsequently, cultured the ZP-free and ZP-enclosed blastomeres in the same well to culture the sister blastomeres together while preventing contact.

### Vital labeling of the sister blastomeres in unsplit two-cell embryos and culture to blastocyst stage

Blastomeres were injected with Oregon Green dextran beads (OGDB) 70 kDa (ThermoFisher cat. no. D7173) and Oyster-568 dextran beads (OY568DB) 70 kDa (Luminartis GmbH Münster, lot no. NC2300B) both at the final concentration of 0.2 microgram/microliter in HCZB medium, using a piezo-driven needle (2–3 micrometers inner diameter, 3–4 micrometers outer diameter). The fluorescently labeled embryos were cultured in KSOM(aa) medium.

### Transcriptome analysis of single blastomeres and single blastocysts

Single blastomeres (from two-cell embryo bisection) and single blastocysts (72 h after two-cell embryo bisection) were lysed and processed for transcriptome analysis. Total RNA was extracted using the ZR RNA Microprep Kit (Zymo Research Corporation, Irvine, USA) without the DNase digestion step. Gene expression profiling was performed using Affymetrix GeneChip® Mouse Transcriptome Array 1.0 (Affymetrix United Kingdom Ltd., High Wycombe, UK) containing 73070 probesets. The fragmented and biotinylated DNA targets were prepared according to the standard Affymetrix WT Pico Reagent Kit protocol (Affymetrix GeneChip® WT Pico Reagent Kit) using 11 amplification cycles from the total RNA starting material available. GeneChips were hybridized, washed and stained in the Affymetrix Fluidics Station 450, according to the standard GeneChip Expression Wash, Stain and Scan protocol (Affymetrix GeneChip Wash, Stain and Scan Kit). Hybridization took place at 45 °C for 16 h. The GeneChips were scanned using the Affymetrix 3000 7 G scanner. The Affymetrix Expression Console and Transcriptome Analysis Console was used for microarray data analysis. The robust multiarray averaging method was applied for background correction, normalization and probe summarization. Gene expression differences were determined by applying an analysis of variance.

### Data availability

In compliance with MIAME guidelines^59^, the data analyzed in this article have been deposited in the NCBI’s Gene Expression Omnibus^60^ and are accessible through GEO Series accession numbers GSE90674 (blastocysts) and GSE94050 (blastomeres).

### Analysis of apoptosis and cell lineage allocation of twin blastocysts

Twin blastocysts were analyzed (72 h after two-cell embryo bisection) by performing an immunostaining followed by confocal microscopy imaging to identify and map the different cell lineages and apoptosis, as described previously^21, 61^. Blastocysts that resulted from two-cell embryos injected with dextran beads were also processed as described previously^21, 61^, except that fixation in paraformaldehyde was protracted to 24 hours.

Rabbit α-cleaved caspase-3 antibodies (Cell Signaling Technology, Leiden, The Netherlands, cat. no. 9661S) were applied to the specimens in a dilution of 1:250 overnight at 4 °C to detect caspase-3 activity. The following primary antibodies were applied simultaneously to the specimens overnight at 4 °C for analysis of cell lineage allocation: anti-Cdx2 mouse IgG1 (Emergo Europe, The Hague, Netherlands, cat. no. CDX2-88), anti-Nanog rabbit IgG (Cosmo Bio, Tokyo, Japan, cat. no. REC-RCAB0002P-F) and anti-Sox17 goat IgG (R&D Systems, cat no. AF1924) in dilutions of 1:200, 1:2,000 and 1:100, respectively. Appropriate Alexa Fluor-tagged secondary antibodies (Invitrogen) were matched to the primaries and incubated for 1–2 h at room temperature.

Blastocysts were placed groupwise in 50 µl drops of PBS on glass slides under coverslips, and imaged using an Olympus BX61 microscope for evaluation of caspase activity. Embryos were placed in 5 µl drops of PBS on a 50-mm thin-bottom plastic dish (Greiner Bio-One, Lumox hydrophilic dish; Frickenhausen, Germany) and overlaid with mineral oil (M8410 Sigma) for analysis of cell lineage allocation. Images were captured on the stage of an inverted microscope (Eclipse 2000-U; Nikon, Düsseldorf, Germany) fitted with a spinning disk confocal unit (Ultra View RS3; Perkin-Elmer LAS, Jügesheim, Germany). A Nikon Plan Fluor 40X oil immersion lens (NA 1.30) was used. Twenty optical sections per blastocyst were captured using a Hamamatsu ORCA ER digital camera (Hamamatsu Photonics KK, Japan). Z-projections were analyzed with ImageJ Version 1.46j.

### Derivation of ES cells from twin blastocysts

Twin blastocysts (72 h after two-cell embryo bisection) were transferred individually onto a feeder layer of γ-ray-inactivated mouse embryonic fibroblasts (C3H background) grown to confluence in 96-well plates (flat bottom) previously. Blastocysts attached to the feeders formed trophoblastic outgrowths on day 3–4 after transfer to feeders. The outgrowths were trypsinized with 0.25% trypsin-EDTA (Gibco, Gaithersburg, MD, USA) and reseeded onto fresh feeder cells on a 96-well plate. Passage zero ES cells (after initial plating of the dissociated outgrowths) were grown for five to six days in culture and further expanded by subsequent passaging (every three days) onto larger well sizes. The ES cell culture media consisted of high-glucose DMEM (Gibco) with 15% fetal bovine serum (BioWest, Nuaillé, France), glutamine and penicillin/streptomycin (Gibco), non-essential amino acids (PAA Laboratories, Pasching, Austria), mercaptoethanol 0.1 mM (Gibco), 1000 Units mL^−1^ LIF (produced in-house), and inhibitors of ERK1/2 and glycogen synthase kinase 3 (CHIR99021 1.5 μM, PD0325901 0.5 μM)^16^.

### Karyotypization

The ES cells were processed for karyotypization, as described previously^62^. Briefly, ES cells were enriched for metaphases by culture in 0.3 μg/ml nocodazole in ES medium for 6 h. Metaphase chromosomes were prepared according to the air-drying method of Tarkowski. Slides were stained with Hoechst 33342 (1 μg/mL) and scored on a Nikon Eclipse TE2000-U fluorescence microscope, as described previously. Only chromosome spreads with ≥40 chromosomes (euploid and hyperaneuploid) were considered, since spreads with less than 40 chromosomes are likely to arise as a preparation artifact. The aneuploidy rate was calculated by dividing the number of spreads with >40 chromosomes by the number of spreads with 40 or more chromosomes.

### Embryo transfer and post-implantation development of MZ twins

Twin blastocysts (72 h after two-cell embryo bisection) were transferred surgically to one uterine horn of pseudopregnant CD1 recipients that had received the copulation plug from vasectomized CD1 males two days prior to the embryo transfer. Prior to surgery, CD1 foster mothers were anesthetized with Ketamin (80 mg/kg)/Xylazin (16 mg/kg)/Tramadol (15 mg/kg) in PBS, delivered intraperitoneally at 10 μl/g body weight. Pregnancies of MZ twins were recorded by caesarean section just prior to term (late embryonic day (E) 18.5).

### Resting metabolic rate of adult MZ twins

This was defined as the rate of oxygen consumption at rest (2 h after beginning the measurements) at 28 °C (within the thermoneutral zone) during the light phase (between 10:00 and 14:00) using the “Chicago method”^63, 64^, which we modified. Briefly, the oxygen consumption of one mouse was measured in a respirometric apparatus consisting of a closed glass chamber (500 mL) with a film of KOH 20 % (w/v) on the ground to absorb the CO_2_ discharged. The amount of oxygen gas in the chamber in this system decreases as the CO_2_ exhaled is bound by KOH. Because the volume of the chamber is constant, the internal pressure decreases, which can be measured using a manometer. The chamber was operated in a water bath set at 28 °C. The manometer was connected to a voltmeter, whereby changes of pressure are detected as changes of voltage expressed in mV. Measurements started when the mouse (weighed) had fully settled down (3 h without food, of which 2 h were in a sedentary state). The oxygen consumed by one mouse per hour per gram of body weight was derived from the time needed to cause a pressure drop of 5 mL in the closed chamber.

### Statistical analysis of developmental rates, cell lineage allocation and gene expression data

Cleavage rates, cell numbers (CDX2, NANOG, SOX17) in the blastocysts and birth rates were analyzed using the statistical program JMP v.13 (SAS). Microarray data analysis was performed in-house using the output of the Affymetrix Expression Console and Transcriptome Analysis Console, exported in Excel format and imported in JMP v.13. Regarding GSEA, gene lists were sorted using the ratio between the higher and the lower mRNA value of the pair (whether blastomere or blastocyst pair) and then imported into the GORILLA^24^.

## Electronic supplementary material

Supplementary Figures S1, S2, S3

Supplementary video

Summary of GSE90674 (blastocysts)

Summary of GSE94050 (blastomeres)
