# Supplementary material for: Totipotency segregates between the sister blastomeres of two-cell stage mouse embryos
Source: Sci Rep. 2017 Aug 15;7:8299. doi: 10.1038/s41598-017-08266-6 (PMC5557898; doi:10.1038/s41598-017-08266-6)
Supplement: Supplementary file 1 — Supplementary Figures S1, S2, S3 [file 41598_2017_8266_MOESM1_ESM.pdf]

Supplementary Figures

Totipotency segregates between the sister blastomeres of two-cell stage mouse embryos

Casser E., Israel S., Witten A., Schulte K., Schlatt S. , Nordhoff V., Boiani M.

Supplementary Figure S1

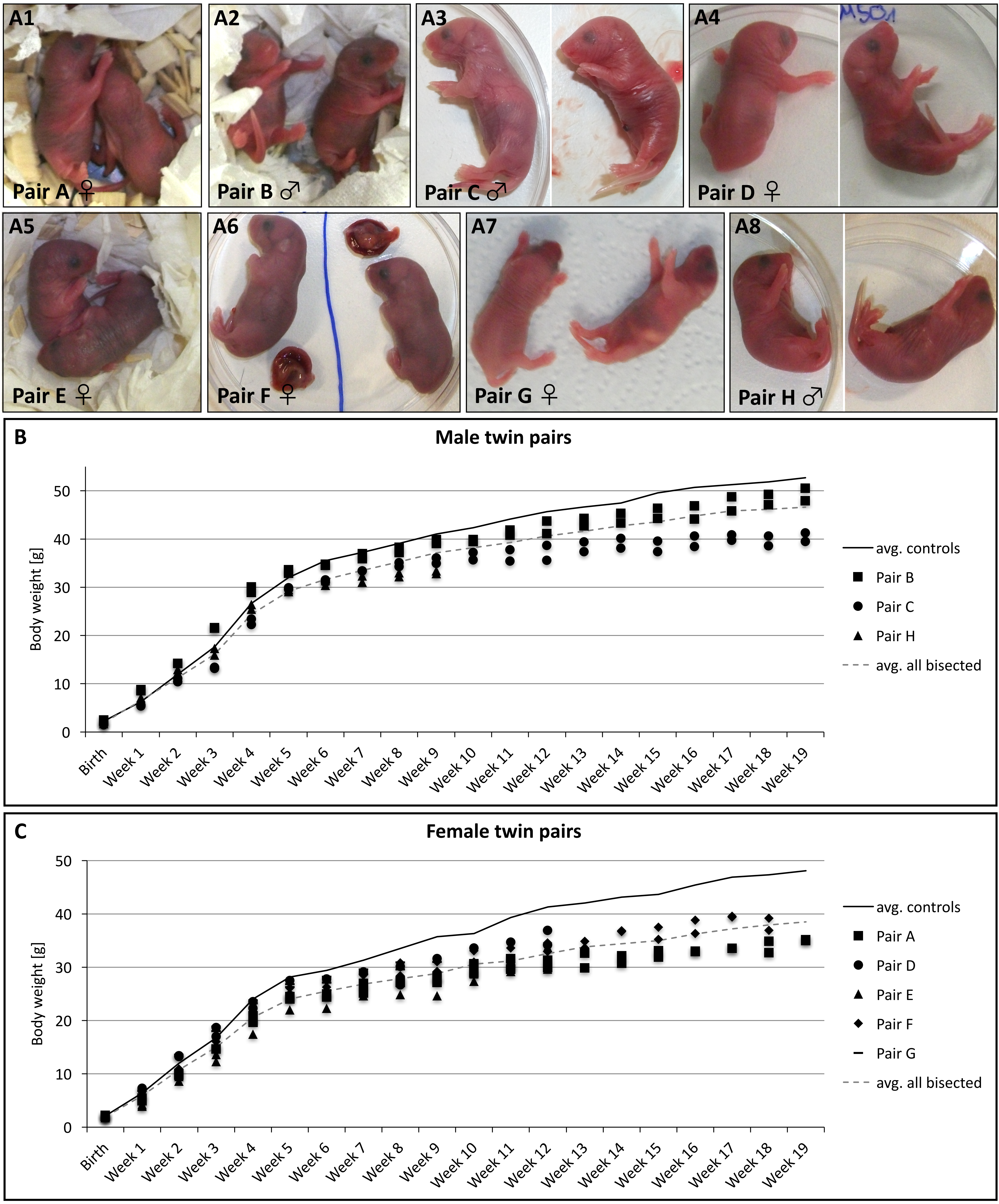

Figure S1. Photographic records of the MZ twin pairs that survived after caesarean section and were characterized further (A). Weight curves of the male (B) and female (C) individuals showing the pair relationships.

Supplementary Figure S2

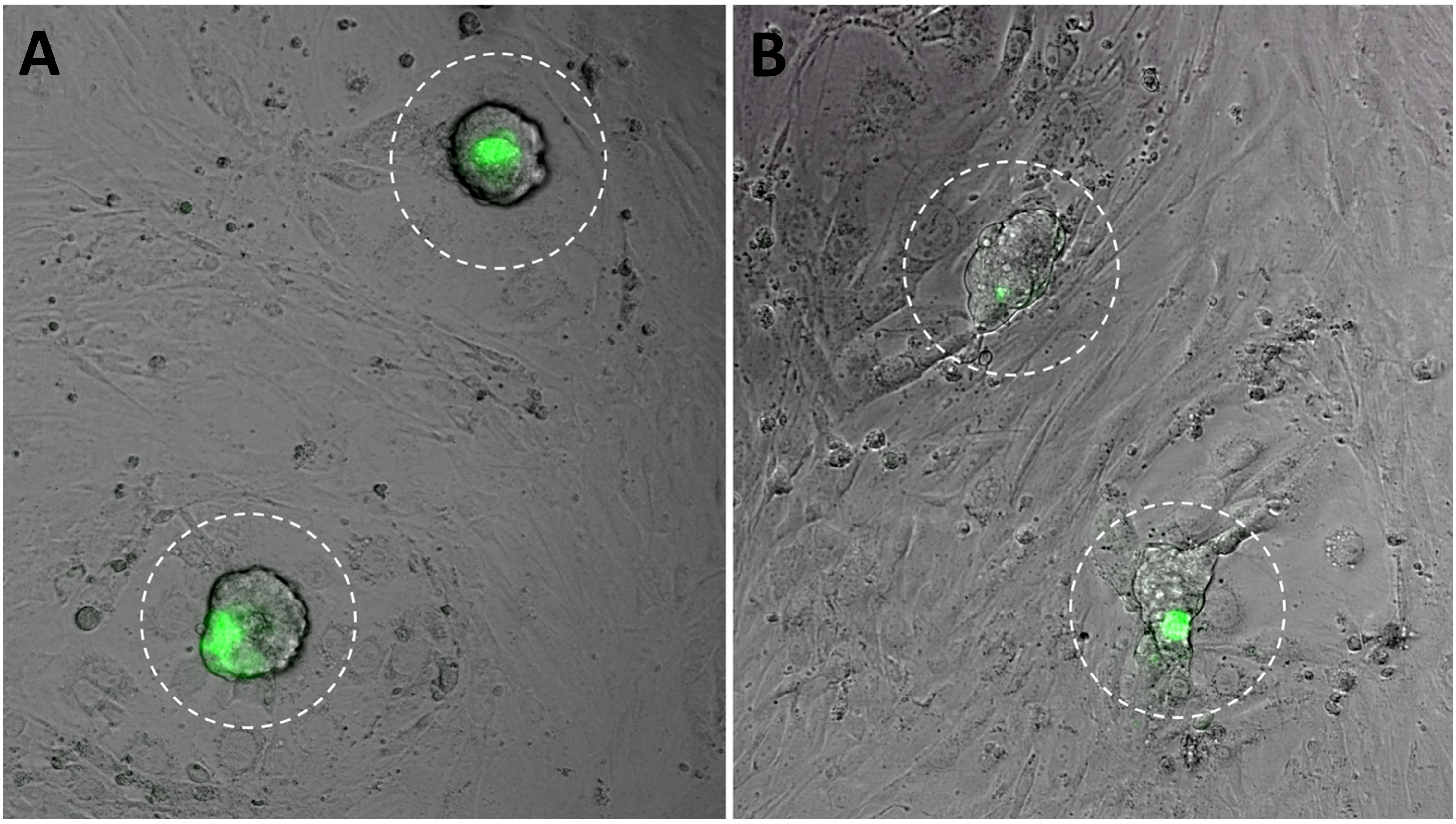

Figure S2. Details of outgrowths with balanced (A) and unbalanced (B) OCT4-GFP positivity in twin and co-twin (original lens magnification 10X)

Supplementary Figure S3

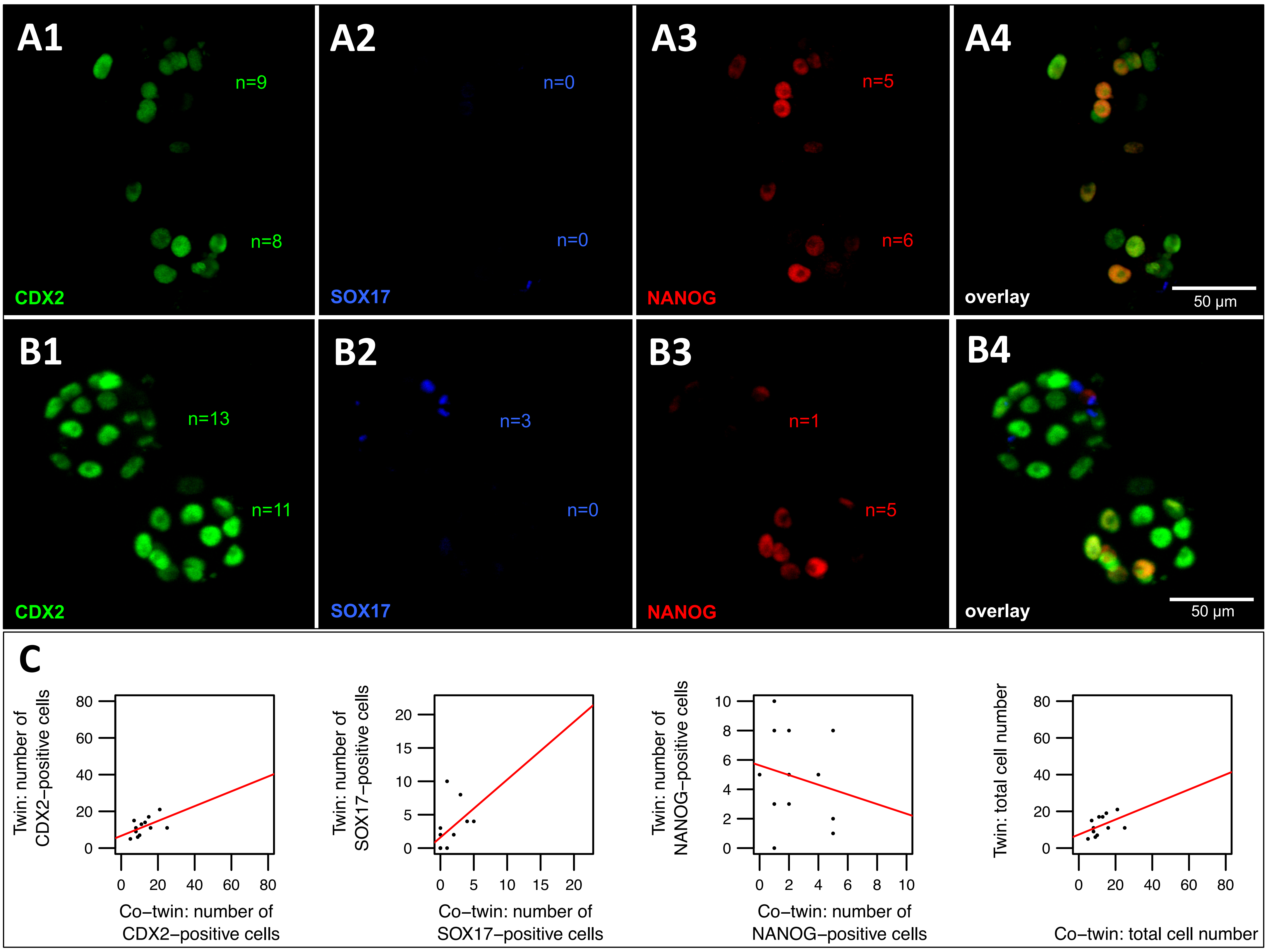

Figure S3. Cell lineage analysis of twin blastocysts produced by splitting of two-cell stage cloned mouse embryos. Immunoconfocal fluorescence images of representative SCNT twin blastocysts, co-stained for cell lineage-specific markers: CDX2 for TE (A1, B1), SOX17 for pEnd (A2, B2) and NANOG for EPI (A3, B3). Microscope objective 20X, 0.75 N.A. Linear correlation analysis (Pearson’s r) of the cells counted in the lineage compartments of each blastocyst pair shows that the main contributor to the discordance was the EPI lineage (C).
